# Transcript profiling of sucrose synthase genes involved in sucrose metabolism among four carrot (*Daucus carota* L.) cultivars reveals distinct patterns

**DOI:** 10.1186/s12870-017-1221-1

**Published:** 2018-01-05

**Authors:** Yan-Jun Liu, Guang-Long Wang, Jing Ma, Zhi-Sheng Xu, Feng Wang, Ai-Sheng Xiong

**Affiliations:** State Key Laboratory of Crop Genetics and Germplasm Enhancement, College of Horticulture, Nanjing Agricultural University, 1 Weigang, Nanjing, 210095 China

**Keywords:** Soluble sugar content, Sucrose synthase, Metabolism, Transcript profiling, *Daucus carota* L

## Abstract

**Background:**

Carrot which contains lots of nutrients has a large demand around the world. The soluble sugar content in fleshy root of carrot directly influences its taste and quality. Sucrose, as an important member of soluble sugar, is the main product of photosynthesis in higher plants and it plays pivotal roles in physiological processes including energy supply, signal transduction, transcriptional regulation, starch and cellulose synthesis, and stress tolerance. Sucrose synthase is a key enzyme involved in sucrose metabolism and is closely related to sucrose content. However, the molecular mechanism involved in sucrose metabolism in carrot has lagged behind.

**Results:**

Here, carrot roots of five developmental stages from four carrot cultivars were collected, and the contents of soluble sugar and sucrose in different stages and cultivars were surveyed. Three *DcSus* genes (*DcSus1*, *DcSus2*, and *DcSus3*), with lengths of 2427 bp, 2454 bp and 2628 bp, respectively, were identified and cloned in carrot. Phylogenetic analysis from the deduced amino acid sequences suggested that three DcSus were clustered into three distinct groups (SUSI, II and III). Results of enzymatic profiles demonstrated that the DcSus activities showed decrease trends during taproot development. Correlation analysis indicated that the DcSus activity showed negative correlation with soluble sugar content and strong negative correlation with sucrose concentration. Quantitative real-time PCR analysis showed that the expression profiles of the *DcSus* genes are significantly different in carrot tissues (root, leaf blade, and petiole), and the expression levels of the *DcSus* genes in the leaf blade were much higher than that in the root and petiole. The expression profiles of *DcSus* genes showed strong negative correlation with both sucrose content and soluble sugar content.

**Conclusions:**

During carrot root development, the soluble sugar content and sucrose content showed increasing trends, while DcSus activities had persisting declinations, which may be due to the decreasing expression levels of genes encoding sucrose synthase. Our data demonstrate that synthesis of sucrose in carrot tissue is closely related with *DcSus* genes. The results from our study would not only provide effective insights of sucrose metabolism in carrot, but also are beneficial for biologists to improve carrot quality.

**Electronic supplementary material:**

The online version of this article (10.1186/s12870-017-1221-1) contains supplementary material, which is available to authorized users.

## Background

As an important nutrient substance, sucrose plays crucial roles in plant growth and development by transferring from “source” to the “sink” organs in the form of assimilated carbon [1]. Sucrose is an important signal molecule in plants that regulates the expression of microRNA, plant hormone and transcription factor, etc. [2–4]. Sucrose also takes part in the biosynthesis of starch [5–10], cellulose [11, 12] and protein. In addition, sucrose participates in plant response to abiotic stresses [13–15].

Previous studies suggested that there are three key enzymes involved in sucrose synthesis and degradation, sucrose-phosphate synthase (EC 2.3.1.14, SPS), sucrose synthase (EC 2.4.1.13, Sus), and invertase (beta-fructofuranosidase, EC 3.2.1.26, Inv). Sucrose-phosphate synthase is involved in sucrose synthesis, while sucrose synthase and invertase are mainly responsible for sucrose break-down [16, 17]. Sucrose is converted into UDPG and fructose under the role of sucrose synthase, which is the first step in the conversion of sucrose to starch [5, 9, 10]. Furthermore, sucrose synthase is also capable of catalyzing sucrose synthesis in a reversible manner.

It is commonly believed that invertase and sucrose synthase participate in the transfer of sucrose to the sink organs, and both of them have been verified to be closely related to the phloem unloading processes [18, 19]. In recent years, studies paid more attention to regulation of the expression patterns of *Sus* genes and enzymology properties of Sus protein, and the activity of Sus have been found to be related to the sink strength in tomato and sweet potato [20, 21]. Usuda et al. found that the sink strength has a strong relationship with the activity of sucrose synthase instead of the activity of invertase [22]. Moreover, the *Sus* genes reported in various plants have been demonstrated to play critical roles in regulation of carbon partitioning, which is responsible for metabolic structure and storage functions of the plant cell [23]. For instance, the validity of Sus cleavage has been verified to be tightly linked with sink strength of different starch storing organs, such as carrot roots, potato tubers, pea embryos and maize kernels [5, 24–27].

Carrot (*Daucus carota* L.), a biennial dicot species of genus *Daucus* (Apiaceae), is widely cultivated as root vegetable all over the world. With its great nutrition and economic value, carrot has been well known as a perfect model plant for genetic and molecular studies [28, 29]. As one of the major sink organs, the growth and development of carrot root requires an increasingly sink activity, which is acquired by activating sucrose metabolism.

To better improve the edible quality of carrot, it is essential to figure out the sucrose metabolism mechanism. In this study, we sampled root materials at five developmental stages from one wild and three cultivated accessions. ‘Songzi’ is a wild type harboring white and lignified root, which was preserved in the National Mid-term Genebank of Vegetable Genetic Resources, Chinese Academy of Agricultural Sciences [30]. For the other three cultivated types, ‘Kurodagosun’ is the most common carrot cultivar with orange and fleshy root, ‘Baiyu’ is a nutrient-rich cultivar with white and fleshy root, and ‘Zizhou’ is a purple carrot cultivar rich in anthocyanins and carotene. The changes in soluble sugar content and sucrose content of four kinds of carrots over the period of root growth were surveyed. The expression of genes involved in sucrose metabolism was also determined by qPCR. The results from our study may provide abundant information for plant breeding and aid in the improvement of carrot nutrition and quality.

## Results

### Growth analysis of carrot plants at various developmental stages

**Fig. 1:**
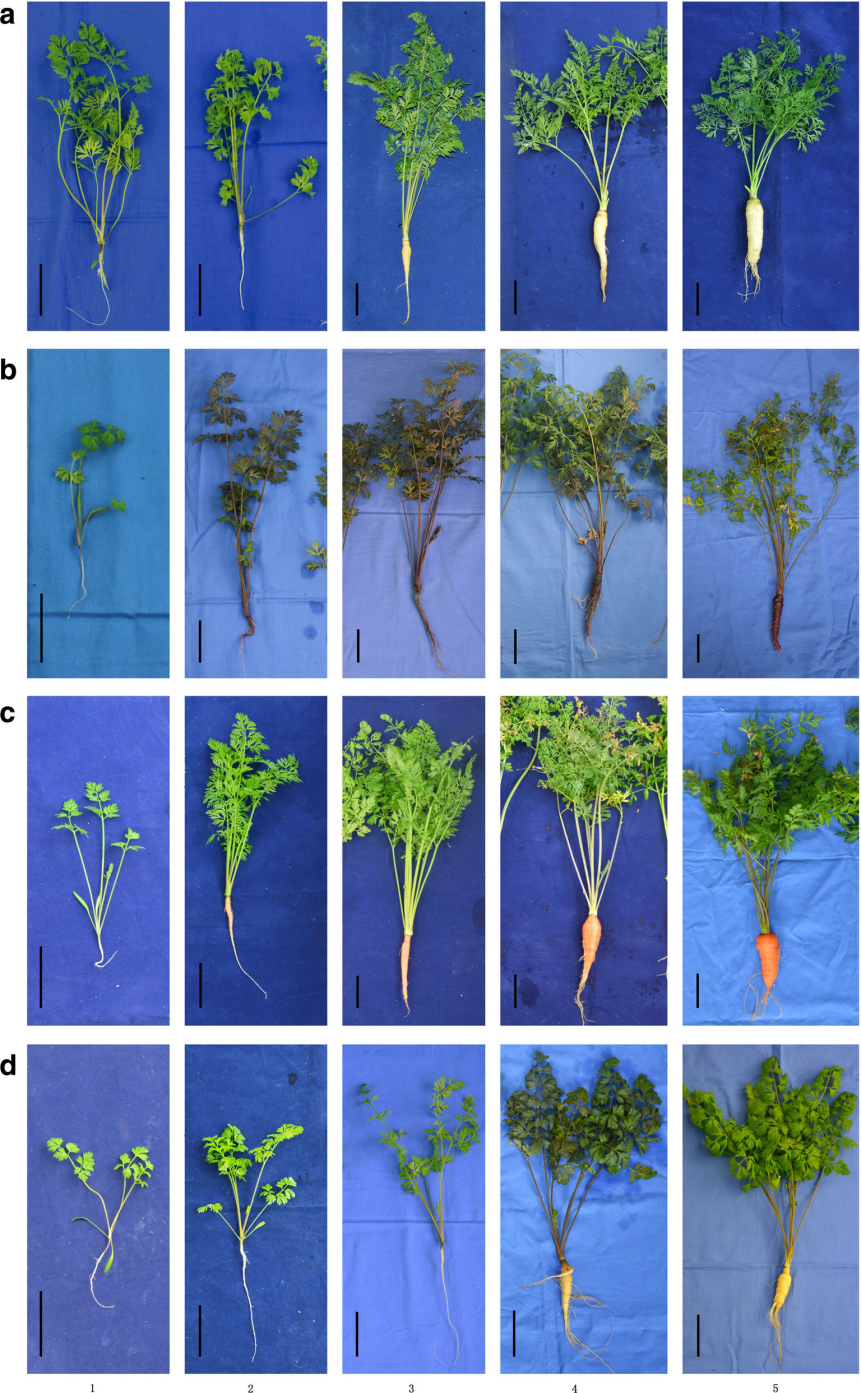
Growth status of four carrot cultivars at five developmental stages. The carrot cultivars used in this study include ‘Baiyu’ (**a**), ‘Zizhou’ (**b**), ‘Kurodagosun’ (**c**), and ‘Songzi’ (**d**). The five developmental stages were stage 1 (30 DAS), stage 2 (45 DAS) stage 3 (60 DAS), stage 4 (75 DAS), and stage 5 (90 DAS). Black lines at the lower left corner represent 5 cm

Carrot roots, petioles, and leaf blades from 30-, 45-, 60-, 75-, and 90 DAS were sampled (Fig. 1). At about 45 DAS, a splash of orange firstly appeared on the root surface of ‘Kurodagosun’, which was maintained in the following development process. Carrot root was consistently purple in color in ‘Zizhou’ after 45 DAS. By contrast, the root color of ‘Baiyu’ was always white during root development. Both ‘Baiyu’ and ‘Songzi’ had a lignified root, but the degree of lignification of ‘Baiyu’ was lower than that of ‘Songzi’.

**Fig. 2:**
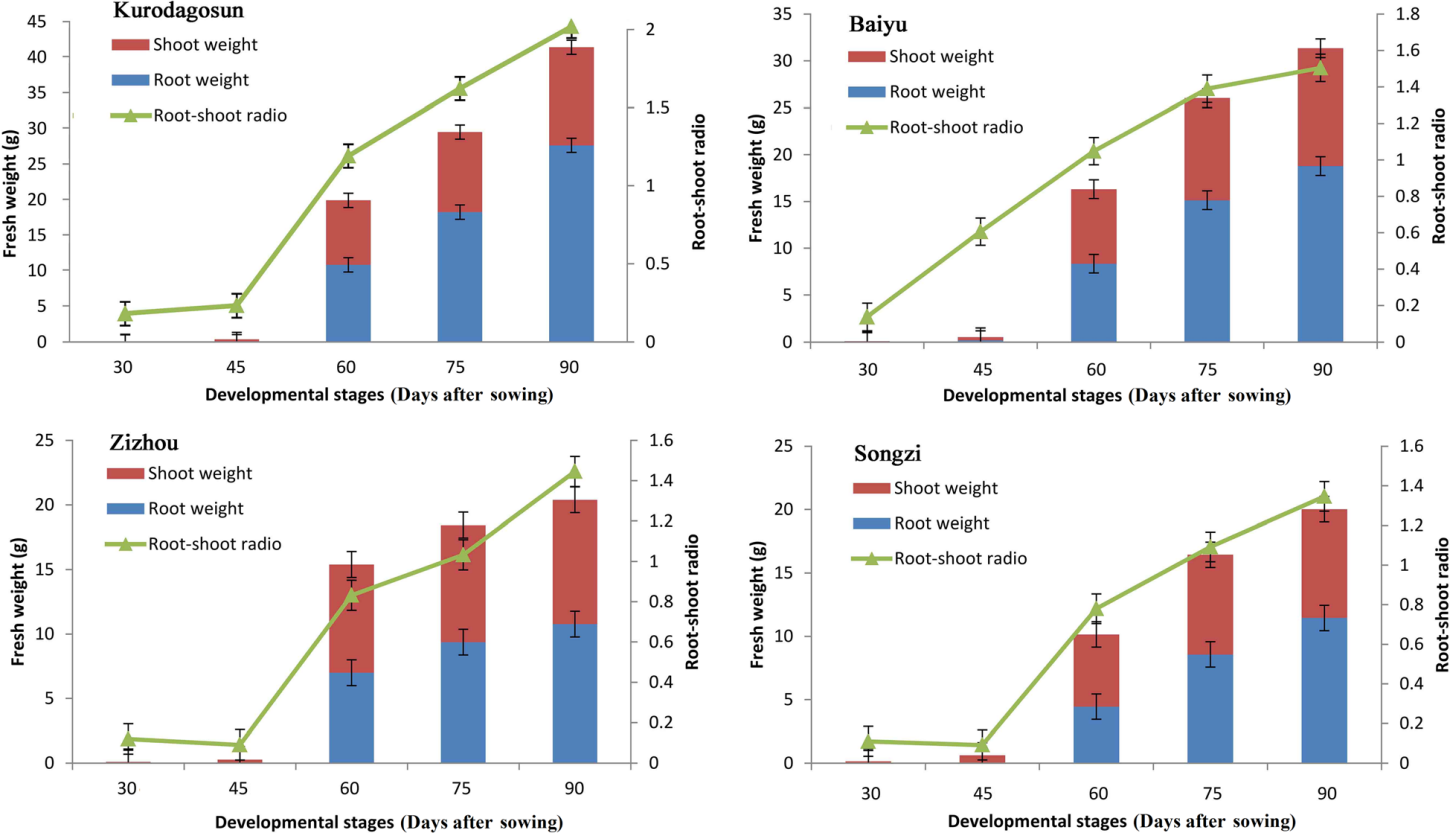
Characteristics of fresh weight of root (blue columns) and shoot (red columns) and root-shoot ratio (green triangles) in carrot. The five developmental stages were stage 1 (30 DAS), stage 2 (45 DAS), stage 3 (60 DAS), stage 4 (75 DAS), and stage 5 (90 DAS). Error bars represent standard deviation among three independent replicates. Data are means of three replicates ± SD

Root development was evaluated by measuring the shoot and root fresh weights (Fig. 2). On the whole, before 45 DAS, the root weight was less than the shoot weight, whereas an opposite trend was shown after then. Both root weight and diameter increased significantly between 45 and 60 DAS, as well as the root-shoot ratio(R/S). After 60 DAS, all cultivars showed a moderate increase in root and shoot weight (Fig. 2).

### Cloning and phylogenetic analysis of genes encoding Sus in carrot

**Fig. 3:**
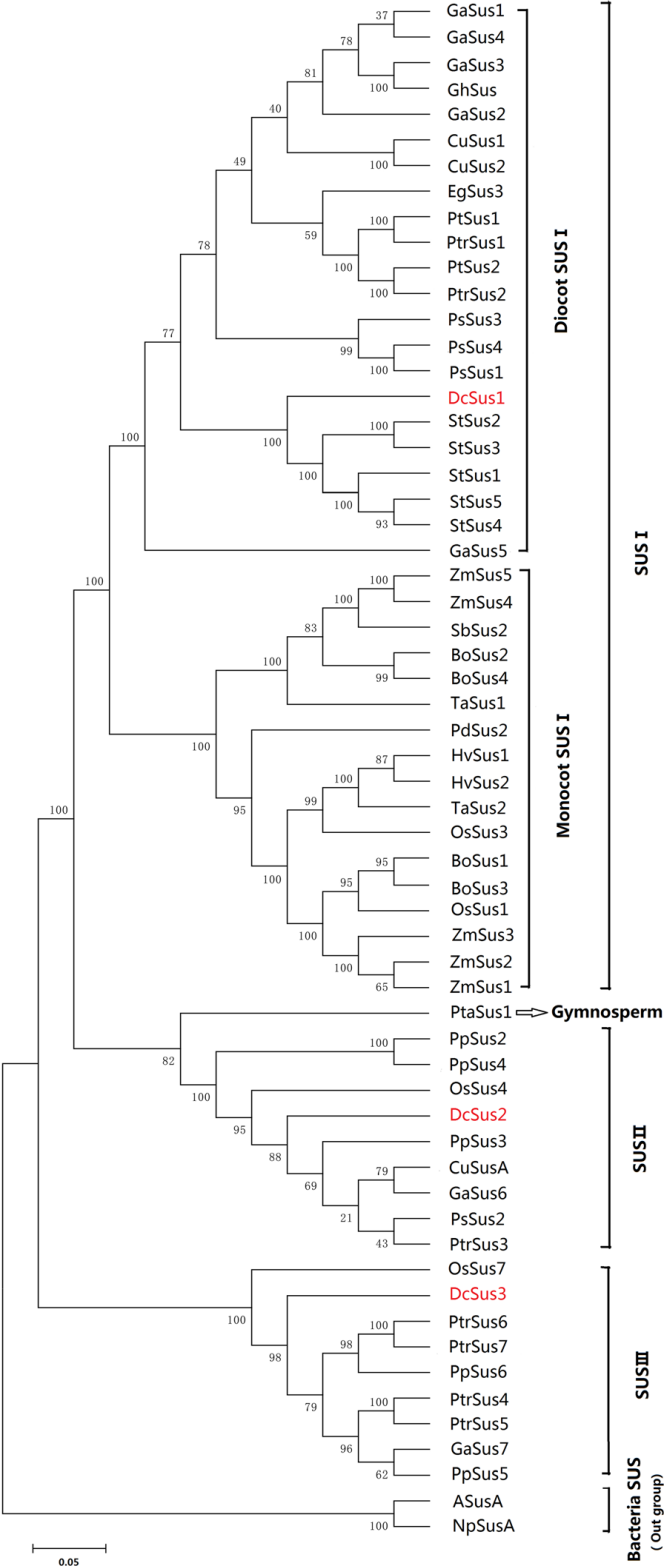
Phylogenetic tree of Sus from carrot and other plant species. Unrooted phylogenetic tree of plant Sus proteins constructed using the neighbor-joining method with the MEGA 6.0 program. The accession numbers of *Sus* genes and their corresponding plant species are listed in Additional file 4

Three genes (*DcSus1*, *DcSus2*, and *DcSus3*) encoding Sus were identified and cloned in carrot roots based on the carrot genomic and transcriptomic database [31]. The primers used for cloning genes were shown in Table 1 and the results were summarized in Additional files 1, 2, and 3. The length of *DcSus1*, *DcSus2*, and *DcSus3* were 2427 bp, 2454 bp and 2628 bp, encoding 808, 817 and 885 amino acids, respectively. To further study DcSus in carrot and investigate the relationship of Sus between plant species, the multiple amino acid alignment of 58 sequences from NCBI database (Additional file 4) and 3 *DcSus* genes were used to construct phylogenetic tree (Fig. 3). With two bacterial Sus used as out-groups, 58 plant Sus genes were classified into three major groups, named Sus I, II and III. As expected, DcSus1, DcSus2 and DcSus3 fall into Sus I, II and III group, respectively. Sus genes from dicots and monocots are found in all the three groups, indicating their evolutionary divergence before the separation of dicots and monocots. In contrast to groups Sus II and III, group Sus I could be further divided into two distinct sub-groups, containing one monocot group and one dicot group.

**Table 1:** Primer sequences used for PCR and qPCR

Gene	ORF (bp)	PCR		qPCR	
		Forward primer (5′-3′)	Reverse primer (5′-3′)	Forward primer (5′-3′)	Reverse primer (5′-3′)
*DcSus1*	2427	CGTTTCATTCGTCCCCTCCGTTCA	CCAGCCCAATGCCTCTTCTTCTC	GGCTCCACAACTACAATGGCAAGA	CAGTAACACGCTCCGCAGTATCAC
*DcSus2*	2454	GAGTGACAGCGAAAGAAGAAGAA	CTCCCATCATATTTGAACAATCCAG	CCTGTATAGAGTTGTCCACGGCATAGA	TGCTCCTCATTCTGCTCTGGATCATAT
*DcSus3*	2628	GGCATTTTATTCTATACGCCCTTA	CAGCTTTGGTTGTCTTCATCCTC	TACTTCTTGACATCCTTCAGGCTCCT	AGGCAGACCTAATACATTCGCTTGG
*DcActin*				CGGTATTGTGTTGGACTCTGGTGAT	CAGCAAGGTCAAGACGGAGTATGG
*DcEF-1α*				TCAAGGATCTCAAGCGTGGTTATGT	CAGCAATGTGGCAAGTGTGACAAT

### Changes of soluble sugar content, sucrose content and sucrose synthase (Sus) activity during root development

**Fig. 4:**
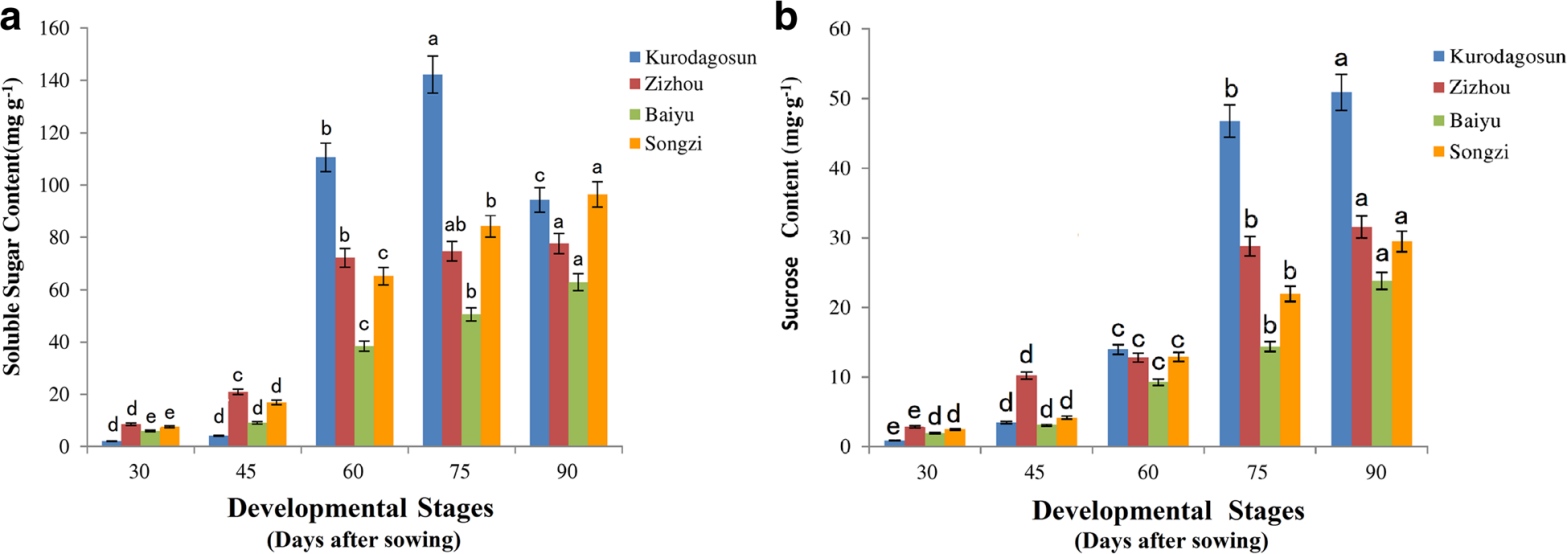
Determination of soluble sugar level and sucrose content in four carrot cultivars at five developmental stages. The soluble sugar content was shown in (**a)** and sucrose content was shown in (**b)**. The five developmental stages were stage 1 (30 DAS), stage 2 (45 DAS), stage 3 (60 DAS), stage 4 (75 DAS), and stage 5 (90 DAS). Error bars represent standard deviation among three independent replicates. Data are means of three replicates ± SD. Different lowercase letters indicate significant differences at *P* < 0.05

**Fig. 5:**
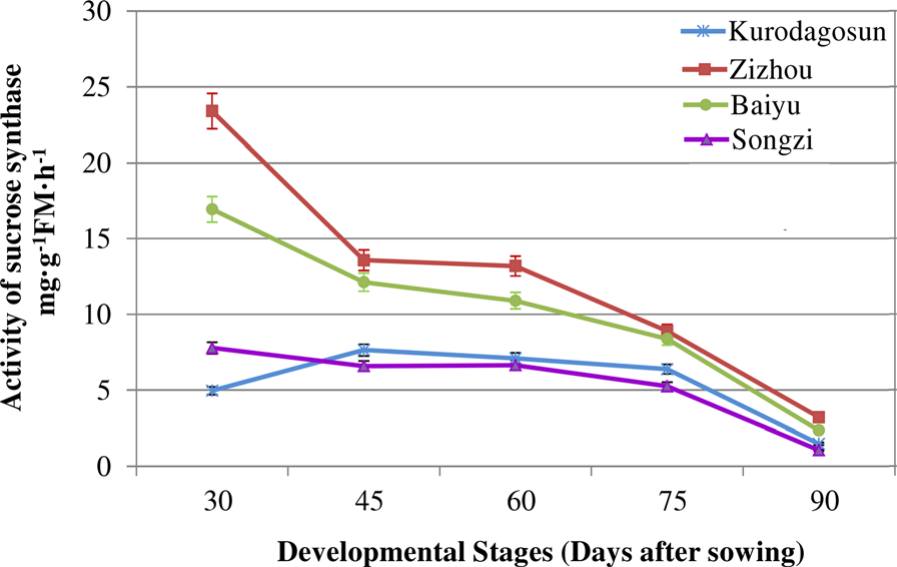
Changes in the activities of DcSus enzymes in carrot during taproot development. Error bars represent standard deviation among three independent replicates. Data are means of three replicates ± SD

**Fig. 6:**
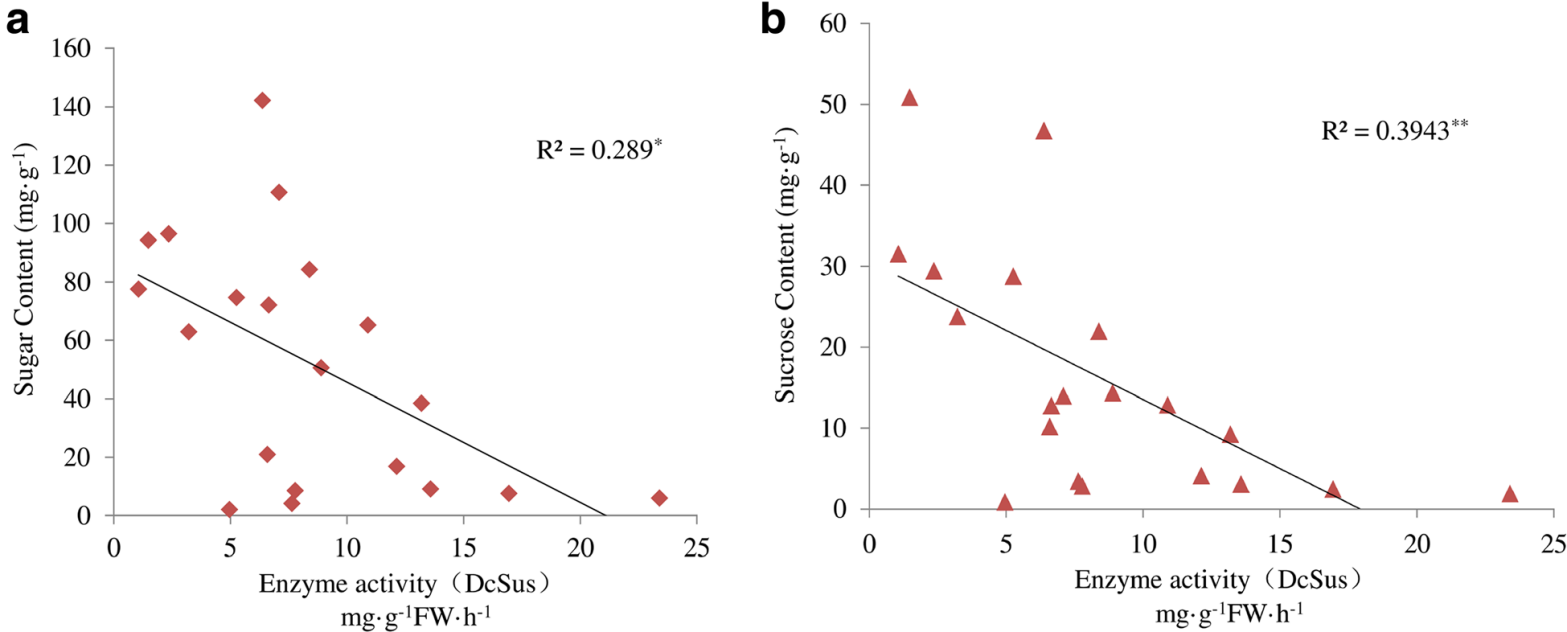
Correlations between specific activities of enzymes DcSus and sucrose concentration and soluble sugar content at different physiological stages of carrot taproot development. *R*^*2*^: Coefficient of determination. **a** Relationship between DcSus enzyme activity and soluble sugar content. **b** Relationship between DcSus enzyme activity and sucrose concentration

To compare the difference in sugar accumulation among the four carrot cultivars, carrot roots at different developmental stages were harvested. The soluble sugar contents were measured at five successive developmental stages (Fig. 4a). Soluble sugar content in ‘Baiyu’, ‘Songzi’, and ‘Zizhou’ roots showed an increasing trend during carrot root development. The soluble sugar content in ‘Kurodagosun’ increased at the first four stages, whereas a decline was detected at the last stage. The soluble sugar content of ‘Zizhou’ was the highest among the four carrot cultivars before 45 DAS, but was surpassed by ‘Kurodagosun’ after then. For the two cultivars with lignified roots, ‘Baiyu’ and ‘Songzi’, the soluble sugar content of the former was always lower than that of the latter (Fig. 4a). On the other hand, the concentration of sucrose increased gradually from 30 DAS to 90 DAS (Fig. 4b). DcSus activity was less in high sucrose accumulating cultivars (‘Kurodagosun’, ‘Songzi’) than in low sucrose accumulating cultivars (‘Baiyu’, ‘Zizhou’). DcSus activity was higher in immature taproots than that in mature taproots in all cultivars. However, the enzymatic activity of sucrose synthases in taproots showed decreasing trends at five developmental stages in four carrot cultivars (Fig. 5). The correlations of DcSus activity with sucrose content and soluble sugar content are depicted in Fig. 6. The DcSus activity showed negative correlation with soluble sugar content (*p* < 0.05; *r* = −0.538) and strong negative correlation with sucrose concentration (*p* < 0.01; *r* = −0.628) (Table 2).

**Table 2:** Correlations between DcSus activity and soluble sugar content and sucrose content

	Enzyme activity	Sucrose content	Enzyme activity	Soluble sugar content
Correlation Coefficient	1	−.628^**^	1	−.538^*^
Sig.(2-tailed)		.003		.014
Square and cross product	568.037	−969.924	568.037	−2336.673
Covariance	29.897	−51.049	29.897	−122.983

Correlations were determined by Pearson correlation coefficient (*r*) analysis. ^*^ indicates significance at *p* < 0.05 and ^**^ indicates significance at *p* < 0.01, respectively

### Expression profiles of genes encoding DcSus in carrot

**Fig. 7:**
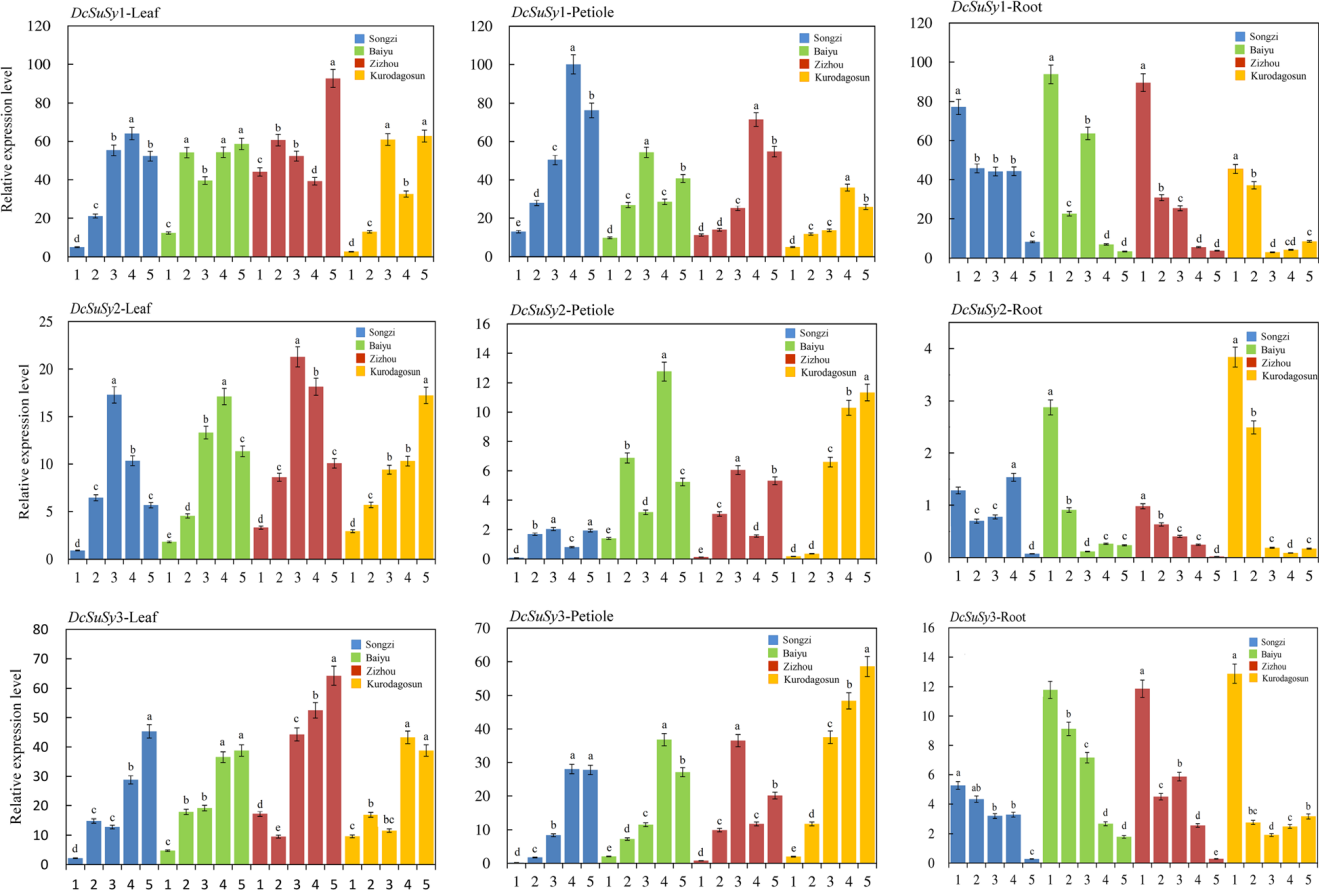
Expression profiles of genes encoding Sus in four kinds of carrots at five developmental stages.The five developmental stages were stage 1 (30 DAS), stage 2 (45 DAS), stage 3 (60 DAS), stage 4 (75 DAS), and stage 5 (90 DAS). Error bars represent standard deviation among three independent replicates. Data indicate mean ± SD of three replicates

**Fig. 8:**
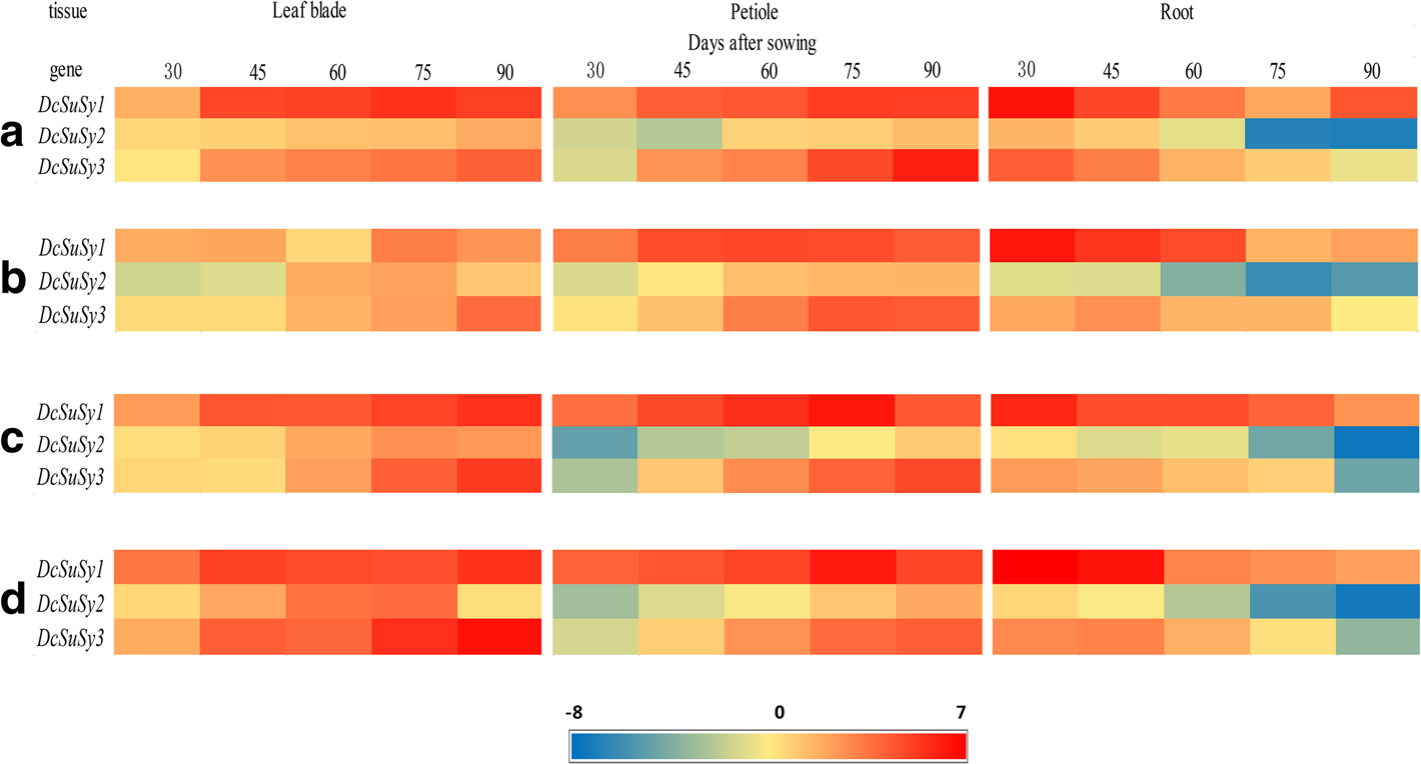
Heat map of the genes encoding sucrose synthase in four carrot cultivars. Cluster analysis was conducted based on average linkage clustering. Each spot was normalized by the log_2_ transformed to represent color scores. Red, yellow, and blue bars represent genes with high, low, and moderate expression, respectively. The four carrot cultivars were ‘Kurodagosun’ (**a**), ‘Baiyu’ (**b**), ‘Songzi’ (**c**), and ‘Zizhou’ (**d**)

qPCR was conducted to detect the relative expression levels of three *DcSus* genes (*DcSus1*, *DcSus2*, and *DcSus3*) at five developmental stages among the four carrot cultivars (Fig. 7). The expression patterns were also compared and plotted in the heat map (Fig. 8). The genes were down-regulated during root growth, which were negatively correlated with sucrose concentration as investigated in the present work. On the contrary, most of the *DcSus* genes were up-regulated during the development of leaf blade and petiole. Among the three *DcSus* genes, the expression profile of *DcSus1* was the highest, whereas relative transcript level of *DcSus2* was the lowest (Fig. 7). The expression profiles of *DcSus* genes were significantly different in different carrot tissues (root, leaf blade, and petiole). Transcript levels of *DcSus1* genes were highest in the petiole, while *DcSus2* and *DcSus3* had the highest level of expression in the leaf blade. The *DcSus* genes were down-regulated throughout the entire process of carrot taproot development, whereas opposite trends were demonstrated in the leaf blade and petiole (Figs. 7 and 8). Correlation analysis were conducted between *DcSus* expression profiles and sucrose and soluble sugar content, the results showed that *DcSus1–3* expression profiles exhibited significant negative correlation with sucrose content (*p* < 0.01; *r* = −.688, −.629, −.675, respectively) and soluble sugar content (*p* < 0.01; *r* = −0.669, −0.572, −0.622, respectively) (Table 3).

**Table 3:** Correlations between the genes of *DcSus* expression levels and sucrose content and soluble sugar content

	Sucrose content	*DcSus1* expression level	*DcSus2* expression level	*DcSus3* expression level
Correlation Coefficient	1	−.688^**^	−.629^**^	−.675^**^
Sig.(2-tailed)		.001	.003	.001
	Soluble sugar content	*DcSus1* expression level	*DcSus2* expression level	*DcSus3* expression level
Correlation Coefficient	1	−.669^**^	−.572^**^	−.622^**^
Sig.(2-tailed)		.001	0.008	.003

Correlations were determined by Pearson correlation coefficient (*r*) analysis. ^*^ indicates significance at *p* < 0.05 and ^**^ indicates significance at *p* < 0.01, respectively

## Discussion

Sucrose biosynthesis and degradation has attracted increasing attention from biologists over the past decades. Plentiful studies have recognized sucrose as a vital metabolite and signaling molecule in response to plant growth and development. Much information on sucrose metabolism has been achieved in plants, such as maize, radish, and sweet potato [21, 32, 33]. However, despite its great economic and nutritional value, very little is known about the sucrose metabolism in carrot.

Accurately identifying and subsequently characterizing the genes involved in sucrose metabolism is a key step towards clarifying their physiological function and metabolic mechanism of different development processes [34]. Recent findings have indicated that most plant species had at least three *Sus* genes encoding Sus [35]. In maize, apart from two tissue-specific *Sus* genes, *Sus1* and *Sh1*, a third *Sus* gene, *Sus3*, which is more similar to dicot than to monocot, has been reported [36]. *Sus1* has a dominant role in starch synthesis and is widely expressed in almost all tissues, while *Sh1* plays a major role in cell wall synthesis and is most highly expressed during the development of endosperm [37]. Till now, to our knowledge, only two genes encoding sucrose synthase had been identified in carrot [38]. Our present work through database searching and molecular cloning indicated carrot *Sus* gene family harbored at least three genes (*DcSus1*, *DcSus2*, and *DcSus3*). Similar to sucrose synthase (*Sus*) genes showing tissue specificity in maize, those from rice, *Lotus japonicus* and *citrus* have been proved to be expressed in stage-dependent and tissue-specific patterns [39, 40]. The same patterns were also found in carrot of this study.

Soluble sugar content showed a continuous increase during five developmental stages in carrot. And the soluble sugar contents among the four carrot cultivars showed significantly difference. We also observed that the sugar levels in different colored carrots are as follows: orange > white > purple, which is consistent with the conclusions from a previous study [41]. Sucrose content had the same variation trends comparing to soluble sugar content. Although Sus can also synthesize sucrose, the degradation reaction dominates in vivo [42, 43]. Sus activity was found to be negatively correlated with sucrose concentration in both high and low sucrose cultivars. The finding was corresponding to Lingle et al.’s research [44]. It is possible that carrot taproot obtain energy via utilizing sucrose as a carbon source and depends on it cleavage into hexose sugars [38].

In the present study, we identified three genes encoding sucrose synthase and demonstrated that *DcSus1* had the highest transcript level and was most abundantly expressed in all the tissues. Although soluble sugar content showed a sustainable growth, the expression levels of *DcSus* genes showed a continuous decline, which were negatively correlated with sucrose accumulation. Similar results were also observed in switch grass, of which, *PvSUS1* was expressed ubiquitously in all the tissues and the soluble sugar content was decreased in *PvSUS1*-overexpressing transgenic plants [45]. The results suggested sucrose synthase may play significant roles in sucrose degradation in carrot.

## Conclusions

The objective of the present study was to examine the relation among sucrose content,enzyme activities and transcript expression of the key enzymes DcSus. In this study, soluble sugar contents of four different cultivated and wild type carrots at five development stages were measured. Soluble sugar content and sucrose concentration showed increasing trends during root development, which may be influenced by the down-regulation of *DcSus* genes involved in sucrose metabolism. DcSus activity was higher in immature taproots than in mature taproots in all cultivars, and the transcript expression of *DcSus* showed a similar pattern. These results will provide useful sources for regulation of sucrose metabolism in carrot.

## Methods

### Plant material and growth conditions

The carrot roots used in this study were obtained from four carrot cultivars (‘Kurodagosun’, ‘Baiyu’, ‘Songzi’, ‘Zizhou’) cultivated in a climate chamber of State Key Laboratory of Crop Genetics and Germplasm Enhancement, Nanjing Agricultural University (32°04′N, 118°85′E). Carrot seedlings were cultivated in flower-pots filled with 1:1 mixture of organic soil and vermiculite. The growth conditions were maintained at light (14 h light/10 h dark) and temperate (25 °C light/18 °C dark) with 60 to 70% relative humidity and 240 μmol m^−2^ s^−1^ light intensity. Carrot tissues (roots, petioles, and leaf blades) were harvested at 30, 45, 60, 75, and 90 days after sowing (DAS) and were frozen immediately in liquid nitrogen and stored at −80 °C until RNA was extracted.

### RNA preparation and cDNA synthesis

Total RNA was isolated from frozen tissues (carrot root, leaf blade and petiole) using an RNA extraction kit (Tiangen, Beijing, China) according to the manufacturer’s protocol. The quantity and quality of total RNA samples were examined by a Nanodrop ND-1000 spectrophotometer (Nanodrop Technologies Inc., Delaware, USA). The integrity of the isolated total RNA was checked using agarose gel electrophoresis (Additional file 5), and genomic DNA was removed using a RT reagent Kit with gDNA Eraser (Perfect Real Time) (TaKaRa, Dalian, China). The cDNA was synthesized from 500 ng of RNA using the PrimeScript RT reagent kit (TaKaRa, Dalian, China) following the manufacturer’s instructions.

### Gene cloning, sequencing and phylogenetic analysis

Genes encoding sucrose synthase of carrot were identified from the carrot genomic and transcriptomic database [31]. Genes were cloned by PCR amplification, which was performed with the following procedures: 94 °C for 5 min; followed by 35 cycles of 94 °C for 30 s, 54 °C for 30 s, 72 °C for 2 min; 72 °C for 10 min. The amplified fragments were inserted into the pMD19-T simple vector (Takara, Dalian, China), and then transformed into the *Escherichia coli* strain (DH5α). The plasmid DNA was sequenced by GenScript Inc. (Nanjing, China). The primers used for PCR amplification are listed in Table 1 and the sequences of the 3 genes encoding sucrose synthase in carrot were shown in Additional files 1-3.

To further investigate the DcSus from carrot, sequence searches were performed using the known DcSus (GenBank No. XM_017367066) as a query to identify homologs of other plants by NCBI blast analysis (https://blast.ncbi.nlm.nih.gov/Blast.cgi). We used MEGA 6.0 software (http://www.megasoftware.net/) to build the neighbor-joining tree from the sequence alignment using following parameters: p-distance model, pairwise gap deletion and 1000 bootstraps. Gene or protein accession numbers of Sus from other plants used in this study are listed in Additional file 4.

### Determination of soluble sugar content and sucrose content

Soluble sugar was purified and measured by the anthrone colorimetric method [46]. In detail, approximately 1 g root tissues were firstly cut into small pieces and ground in a mortar with liquid nitrogen. Then 10 mL of distilled water was pipetted into the sample and incubation with boiling water for 60 min. Subsequently, the supernatant gathered by centrifugation at 12,000×*g* for 20 min at 4 °C was diluted to 25 mL with distilled water. Then, 0.5 mL of mixture was collected and added with 1.5 mL of distilled water, 0.5 ml of anthrone ethyl acetate reagent, and 5 mL of concentrated sulfuric acid. After cooling, absorbance was measured at 620 nm. We used a mix of 5 mL of concentrated sulfuric acid and 0.5 mL of anthrone ethyl acetate reagent as blank control. Finally, sucrose calibration curves were constructed for accurate quantification.

Sucrose was determined by the anthrone method [47]: 70 μL of reaction solution was added to 70 μL of 30% KOH, boiled for 10 min, and cooled to room temperature; 1 m L anthrone reagent (0. 15 g anthrone in 100 ml 76% [*v*/v] sulfuric acid) was added and the reaction was incubated at 37 °C for 20 min. A650 was measured immediately. The experiments were repeated with three independent samples.

### Extraction and assay of enzymes

Enzymes activity of DcSus was determined from carrot roots. Freeze-dried materials (1 g) were homogenized in 100 mM HEPES/KOH (pH 7.5) that contained 5 mM MgCl_2_, 1 mM EDTA, 0.1% *β*-mercaptoethanol and 0.1% Triton X-100. The homogenate was centrifuged at 18,000 rpm for 20 min at 4 °C, and the supernatant was desalted on a HiTrap desalting column (GE Healthcare Life Sciences, Beijing, China).

### Assay of sucrose synthase (Sus)

Sucrose synthase in cleavage direction was measured as the formation of UDPGIc according to Zrenner et al. [48], with slight modifications: The assay mixture contained 20 mM HEPES/KOH (pH 7.5), 100 mM sucrose, 4 mM UDP and 5 mM DTT. 50 μL of the desalted homogenate was added to 50 μL of the mixture. The reaction proceeded for 20 min at 34 °C, and was stopped by boiling for 10 min. UDPGIc produced was measured in a subsequent reaction in a 1 ml volume containing 200 mM glycine/HCl (pH 8.5), 5 mM MgCl_2_, 2 mM NAD^+^ and 0.02 U /mL UDP-glucose pyrophosphorylase. Absorbance was measured at 340 nm. Control assays were performed without sucrose and without UDP.

### Gene expression analysis by qPCR

Total RNA (1 μg) from each sample of different treatments was reverse transcribed using the PrimeScript 1st Strand cDNA Synthesis Kit (TaKaRa, Dalian, China). Primers used for qPCR were designed with Primer Premier 6 software (Table 1) spanning at least one intron, and a melting curve was performed to verify the specificity of primer amplification (Additional file 6). To determine the amplification efficiency for each primer set, the calibration curve for each gene was obtained by performing real-time PCR with four dilutions of cDNA (10 × , 10^2^ × , 10^3^ × , 10^4^×) (Additional file 7). Using Bio-Rad IQ5 real-time PCR System (Bio-Rad, CA, USA), qPCR was conducted with SYBR Premix *Ex Taq* (TaKaRa, Dalian, China). Each reaction contained 2 μL of 10 × diluted cDNA strand, 10 μL of SYBR Premix *Ex Taq*, 7.2 μL of deionized water, and 0.4 μL of each primer (20 mM), accumulating a final volume of 20 μL. PCR was strictly performed according to the following standards: 95 °C for 30 s, followed by 40 cycles of 95 °C for 5 s and 60 °C for 30 s. The specificity of the individual PCR amplification was checked using a heat dissociation protocol from 65 to 95 °C following the final cycle of the PCR. The results were normalized against the carrot reference genes, *DcActin* and *DcEF1-α*, which were verified to exhibit stable levels of expression in a broad range of carrot tissues [49]. Relative gene expression level was calculated by the 2^−ΔΔCT^ method [50]. Analysis was conducted on the data from three independent reactions (technical replicates) using samples from three biological replicates. The data used in this manuscript mainly came from our group’s previous RNA-seq transcriptomic database [31, 51–53].

### Statistical analysis

Statistical analysis was performed using One-way ANOVA on SPSS Version 20.0, followed by Duncan’s multiple range test. Significant differences were set at *P* < 0.05 for all analyses. Correlation between enzyme activity and sucrose content and soluble sugar content, *DcSus* expressions levels and sucrose content and soluble sugar content were analyzed using Pearson correlation coefficient (*r*) at significance levels *p* < 0.01 and *p* < 0.05.

## Additional files

Additional file 1: Figure S1. Nucleotide acid and deduced amino acid sequences of DcSus1 from carrot (DOC 33 kb)

Additional file 2: Figure S2. Nucleotide acid and deduced amino acid sequences of DcSus2 from carrot (DOC 33 kb)

Additional file 3: Figure S3. Nucleotide acid and deduced amino acid sequences of DcSus3 from carrot (DOC 34 kb)

Additional file 4: Table S1 List of sucrose synthase gene sequences used in this study. (DOC 37 kb)

Additional file 5: Figure S4. Results of RNA Electrophoresis. DC27 represents ‘Kurodagosun’; BY represents ‘Baiyu’; DC25 represents ‘Zizhou’; SZ represents ‘Songzi’. (DOC 379 kb)

Additional file 6: Figure S5. Melting curves of DcSus genes related encoding sucrose synthase and two reference genes using in qPCR. (DOC 188 kb)

Additional file 7: Figure S6. Standard curves for DcEF1-α, DcActin, DcSus1, DcSus2 and DcSus3. The linear correlation (R2) and PCR efficiencies (% E = (10[−1/slope] - 1) × 100%) were calculated from the standard curve. (DOC 1194 kb)

## Abbreviations

DAS: Days after sowing
Inv: Beta-fructofuranosidase
qPCR: Quantitative real-time polymerase chain reaction
SPS: Sucrose-phosphate synthase
Sus: Sucrose synthase
