# Supplementary material for: Transcript profiling of sucrose synthase genes involved in sucrose metabolism among four carrot (Daucus carota L.) cultivars reveals distinct patterns
Source: BMC Plant Biol. 2018 Jan 5;18:8. doi: 10.1186/s12870-017-1221-1 (PMC5756371; doi:10.1186/s12870-017-1221-1)
Supplement: Supplementary file 1 — Nucleotide acid and deduced amino acid sequences of DcSus1 from carrot (DOC 33 kb) [file 12870_2017_1221_MOESM1_ESM.doc]

**Additional file 1:**

Fig S1: Nucleotide acid and deduced amino acid sequences of *DcSus1* from carrot.

1 ATGGGTGAACCTGTTCTGACCCGAGTTCACAGCCTCCGGGAACGTATGGATTCAACTTTGGCCAATCATCGCAAT

M G E P V L T R V H S L R E R M D S T L A N H R N

76 GAAATCTTGATGTTTCTATCAAGGATTGAAAGTCATGGAAAAGGAATCTTGAAACCTCACCAGCTTCTAGCTGAG

E I L M F L S R I E S H G K G I L K P H Q L L A E

151 TATGAAGCTATCAGCAAAGAAGACAAGCTGAAACTGGACGATGGTCATGGCGCCTTCGCGGAAGTTATTAAGTCT

Y E A I S K E D K L K L D D G H G A F A E V I K S

226 ACGCAGGAAGCCATAGTTTCTCCTCCATGGGTAGCACTTGCTATTCGGCTCAGACCTGGTGTGTGGGAATATGTA

T Q E A I V S P P W V A L A I R L R P G V W E Y V

301 AGAGTCAACGTGCATCACCTTGTCGTCGAGGAATTGAGTGTGCCTCAGTATCTTCAATTTAAAGAGGAGCTTGTC

R V N V H H L V V E E L S V P Q Y L Q F K E E L V

376 ATTGGATCAAGCGATGCAAACTTTGTCCTTGAACTCGATTTTGCACCATTCACCGCCTCTTTCCCTCGTCCAACA

I G S S D A N F V L E L D F A P F T A S F P R P T

451 CTTACCAAGTCTATAGGGAATGGAGTTGAGTTTCTCAACAGGCATCTTTCTGCAAAAATGTTTCATGGCAAGGAC

L T K S I G N G V E F L N R H L S A K M F H G K D

526 AGCATGCACCCACTTCTTGAGTTTCTCCGGCTCCACAACTACAATGGCAAGACCCTGATGCTGAACAACAGAGTT

S M H P L L E F L R L H N Y N G K T L M L N N R V

601 CAGAATGTGAATGGTCTCCAATCTATGCTGAGGAAAGCTGGGGATTATCTCTCTACACTACCATCAGACACACCT

Q N V N G L Q S M L R K A G D Y L S T L P S D T P

676 TACTCTGAATTTGAGCACAAGTTCCAAGAGATTGGTTTTGAGAGGGGTTGGGGTGATACTGCGGAGCGTGTTACT

Y S E F E H K F Q E I G F E R G W G D T A E R V T

751 GAGATGTTTCACATGCTTCTGGACCTTCTTGAGGCCCCTGATGCATCCACTCTTGAGACATTTTTGGGGAAAATT

E M F H M L L D L L E A P D A S T L E T F L G K I

826 CCAATGGTTTTCAATGTTGTTATTCCCTCCCCTCAAGGTTACTTTGCTCAAGAAAATGTTTTGGGACATCCCGAC

P M V F N V V I P S P Q G Y F A Q E N V L G H P D

901 ACTGGTGGCCAGGTTGTCTACATTTTGGATCAAGTTCCTGCATTGGAGCGTGAGATGATTAAGCGCATAAAGGAG

T G G Q V V Y I L D Q V P A L E R E M I K R I K E

976 CAAGGACTCGACATCAAGCCTCGTATTCTGATTGTAACACGCCTTCTGCCGGATGCAGTAGGTACCACTTGCAAT

Q G L D I K P R I L I V T R L L P D A V G T T C N

1051 CAGCGCCTGGAGAAAGTGTTTGGAGCTGAACACGCCCATATTCTTAGAGTCCCCTTTAGAACTGAGAAAGGAATT

Q R L E K V F G A E H A H I L R V P F R T E K G I

1126 TTGCGCAAATGGATCTCCCGCTTTGAAGTTTGGCCCTACATTGAGACTTTCACTGAGGATGTTGCAAAAGAAATT

L R K W I S R F E V W P Y I E T F T E D V A K E I

1201 GCCTTGGAGTTGCAGGCCAAGCCAGATCTGATTATTGGAAACTATAGCGAGGGTAATCTTGTTGCATCCTTGCTG

A L E L Q A K P D L I I G N Y S E G N L V A S L L

1276 GCACACAAATTAGGTGTTACCCAGTGTACCATTGCTCATGCTTTAGAGAAAACAAAGTACCCTGATTCCGACATC

A H K L G V T Q C T I A H A L E K T K Y P D S D I

1351 TACTGGGAGAAATTTGACAAGAAGTATCACTTTTCCAGTCAGTTTACAGCCGATCTTATTGCAATGAATCATACT

Y W E K F D K K Y H F S S Q F T A D L I A M N H T

1426 GATTTCATCATCACCAGCACATTCCAGGAGATAGCTGGAAGTAAGGATACTGTTGGACAGTATGAGAGTCATACT

D F I I T S T F Q E I A G S K D T V G Q Y E S H T

1501 GCCTTTACAATGCCCGGATTGTACCGAGTTGTGCATGGGATAGATGTCTTCGACCCAAAATTCAACATCGTTTCA

A F T M P G L Y R V V H G I D V F D P K F N I V S

1576 CCAGGTGCAGACACATCTGTTTATTTCTCTTACAAAGAGAAGGAAAAGAGGCTGACAACACTTCACCCTGAAATT

P G A D T S V Y F S Y K E K E K R L T T L H P E I

1651 GAGGAACTTCTTTACAGTTCGGTTGAAAATGAAGAACACCTGTGTATCATAAAAGACAAGAATAAGCCTATACTG

E E L L Y S S V E N E E H L C I I K D K N K P I L

1726 TTCACCATGGCGAGGTTGGACAATGTGAAGAACTTGACAGGATTCGTCGAGTGGTATGCTAAGAGCCCCAAGCTA

F T M A R L D N V K N L T G F V E W Y A K S P K L

1801 CGTGAGTTGGTGAACCTTGTTGTCGTTGGTGGAGACCGTAGGAAGGAATCAAAGGATCTGGAAGAACAAGCACAG

R E L V N L V V V G G D R R K E S K D L E E Q A Q

1876 ATGAAGAAAATGTACGAGCTTATTGATACCTACAAGCTGAATGGTCAATTCCGATGGATTTCTTCTCAGATGAAC

M K K M Y E L I D T Y K L N G Q F R W I S S Q M N

1951 CGTGTGAGGAATGGTGAACTTTATCGTTACATAGCTGACACAAAGGGTGCTTTTGTGCAACCTGCATTCTACGAG

R V R N G E L Y R Y I A D T K G A F V Q P A F Y E

2026 GCTTTTGGTTTAACTGTTGTTGAGGCCATGACCTGTGGATTGCCAACCTTCGCAACTCTCCATGGTGGTCCAGCT

A F G L T V V E A M T C G L P T F A T L H G G P A

2101 GAGATCATTGTTCACGGGAAATCTGGTTTTCATATCGACCCATATCATGGTGAGCAAGTGGCTGAGCTCCTTGTT

E I I V H G K S G F H I D P Y H G E Q V A E L L V

2176 AACTTCTTCGAGAAGTGCAAGACCGATCCTTCTCAATGGGACGCCATTTCAGCAGGCGGCCTTAAACGTATCCAG

N F F E K C K T D P S Q W D A I S A G G L K R I Q

2251 GAGAAATACACTTGGCAAATCTACTCGGAGAGACTATTGACATTGGCTGGGGTTTACGGATTCTGGAAGCATGTC

E K Y T W Q I Y S E R L L T L A G V Y G F W K H V

2326 TCCAAGCTCGATCGCCTTGAGATCCGTCGTTATCTTGAAATGTTTTGTGCTCTTAAGTACCGCAAGTTGGCTGAA

S K L D R L E I R R Y L E M F C A L K Y R K L A E

2401 TCAGTTCCACTTGCTAAAGATGA

S V P L A K D *
