# Supplementary material for: Transcript profiling of sucrose synthase genes involved in sucrose metabolism among four carrot (Daucus carota L.) cultivars reveals distinct patterns
Source: BMC Plant Biol. 2018 Jan 5;18:8. doi: 10.1186/s12870-017-1221-1 (PMC5756371; doi:10.1186/s12870-017-1221-1)
Supplement: Supplementary file 2 — Nucleotide acid and deduced amino acid sequences of DcSus2 from carrot (DOC 33 kb) [file 12870_2017_1221_MOESM2_ESM.doc]

**Additional file 2:**

Fig S2: Nucleotide acid and deduced amino acid sequences of *DcSus2* from carrot.

1 ATGGCAACTCGTGAACTAAAAAAATTACCAAGTATTAGAGATAGGGTAGAAGACACCCTCTCTGCTCACCGCAAT

M A T R E L K K L P S I R D R V E D T L S A H R N

76 GAACTTGTCTCTCTTCTCTCCAGATATGTGGCACAAGGGAAGGGGATATTGCAACCACATCATTTGATAGATGAG

E L V S L L S R Y V A Q G K G I L Q P H H L I D E

151 TTGGAAAGCAGTATAGGTGATGAAGATTCCGGAAAACATCACCTTAGAGATGGTCTTTTTGGCCAAATCCTTCAA

L E S S I G D E D S G K H H L R D G L F G Q I L Q

226 TCAACTCAGGAAGCAATAATTCTGCCCCCGTTTGTAGCAATAGCAGTTCGACCAAGGCCTGGTGTTTGGGAATAT

S T Q E A I I L P P F V A I A V R P R P G V W E Y

301 GTGCGCTTAAATGTGTATGAGCTCAGTGTGGAGCAGTTAGACGTCTCTCAATATCTTCACTTCAAGGAAGAACTT

V R L N V Y E L S V E Q L D V S Q Y L H F K E E L

376 GTTAACGGGCAGATTGATGACAAGTTCATGCTTGAGCTTGATTTTGAGCCATTTAATGCGACTGTCCCACGCCCC

V N G Q I D D K F M L E L D F E P F N A T V P R P

451 ACTCGATCCTCATCCATTGGCAATGGGGTTCAGTTTCTCAACCGTCACCTCTCTTCAATTATGTTCCGAAACAAA

T R S S S I G N G V Q F L N R H L S S I M F R N K

526 GATTGTTTGGAGCCCTTGCTTAATTTTCTTCAAGCACACAATTATAAAGGTCATGTGATGATGATTAATGATCGG

D C L E P L L N F L Q A H N Y K G H V M M I N D R

601 ATACGCTCCATATCGAGACTTGAGTCTGCTTTAACAAAGGCAGAAGATTATCTTTCTAAGCAACTACCAGATGCA

I R S I S R L E S A L T K A E D Y L S K Q L P D A

676 CCATTTTCCCAGTTTGAATATGATTTGCAAGGAATGGGGTTTGAGAAAGGCTGGGGTGATACTGCTGAACGTGTT

P F S Q F E Y D L Q G M G F E K G W G D T A E R V

751 TTGGAAATGATGCATCTTCTTTCTGACATCCTTCAAGCTCCTGATCCTGCCAGCTTAGAGACATTCCTGGGTAGA

L E M M H L L S D I L Q A P D P A S L E T F L G R

826 ATTCCTATGGTATTTAACGTAGTCATTTTATCTGTACATGGATTCTTTGGCCAGGCAAATGTTCTGGGTTTGCCC

I P M V F N V V I L S V H G F F G Q A N V L G L P

901 GACACTGGTGGTCAGATTGTTTACATACTTGATCAAGTACGGGCTTTGGAGAATGAAATGCTTCTCAAGTTGAAG

D T G G Q I V Y I L D Q V R A L E N E M L L K L K

976 CAGCAAGGACTGAATATTACTCCCAGAATTCTAATTGTCACCCGACTTATACCTGATTCAAAGGGTACCACATGC

Q Q G L N I T P R I L I V T R L I P D S K G T T C

1051 AACCAGCGACTGGAAAGAGTTAGTGGGACTGATCACACACATATTTTGCGTGTTCCATTTAGAACAGAGCATGGA

N Q R L E R V S G T D H T H I L R V P F R T E H G

1126 GTCCTTCGTAAGTGGATCTCAAGGTTTGATGTATGGCCCTATCTGGAGAATTTTACTGAGGATGCGGCAAGTGAA

V L R K W I S R F D V W P Y L E N F T E D A A S E

1201 ATTTGTGCTGAATTACAAGGCAATCCAGACCTCATCATTGGCAACTACAGTGATGGCAATCTCGTTGCGTCTCTG

I C A E L Q G N P D L I I G N Y S D G N L V A S L

1276 TTATCTTATAAGATGGGAGTTACACAGTGTACCATCGCTCATGCACTGGAAAAGACGAAATACCCTGATTCGGAC

L S Y K M G V T Q C T I A H A L E K T K Y P D S D

1351 ATATATTGGAAGAAATTTGACGAAAAATATCATTTCTCATGTCAATTTACTGCTGATCTAATAGCCATGAATAAT

I Y W K K F D E K Y H F S C Q F T A D L I A M N N

1426 GCTGATTTCATCATCACAAGCACGTATCAGGAGATCGCTGGAACGAAGGATACTGTTGGTCAGTATGAGAGCCAT

A D F I I T S T Y Q E I A G T K D T V G Q Y E S H

1501 TCATCTTTCACTCTCCCAGGCCTGTATAGAGTTGTCCACGGCATAGATGTTTTTGATCCCAAATTCAATATTGTT

S S F T L P G L Y R V V H G I D V F D P K F N I V

1576 TCCCCTGGGGCCGATATGTGCATATACTTCCCATACTCTGAGAAGGAAAAAAGACTTACGTCCCTACATGGTTCA

S P G A D M C I Y F P Y S E K E K R L T S L H G S

1651 ATTGAGAAGTTGCTATATGATCCAGAGCAGAATGAGGAGCATGTGGGTTCGCTGAGTGATCACTCGAAACCGATG

I E K L L Y D P E Q N E E H V G S L S D H S K P M

1726 ATTTTTTCAATGGCTAGGCTTGATAGGGTCAAAAACATTACAGGTCTAGTAGAGTGCTATGCAAAAAATACTAAG

I F S M A R L D R V K N I T G L V E C Y A K N T K

1801 TTGAGGGAACTGGCAAACCTTGTTATCGTTGCTGGTTACAATGATGTTAAGAAGTCCAATGACAGAGAAGAAGTT

L R E L A N L V I V A G Y N D V K K S N D R E E V

1876 GTAGAAATTCAGAAGATGCATGATCTGATTAAGCAATACAGCTTGGATGGCCAGTTGCGATGGATATCAAGCCAA

V E I Q K M H D L I K Q Y S L D G Q L R W I S S Q

1951 ACAAATAGAGCACGCAATGGGGAGCTTTATCGATACATAGCTGACAAGAGGGGTATCTTCGTACAGCCTGCATTT

T N R A R N G E L Y R Y I A D K R G I F V Q P A F

2026 TATGAAGCCTTTGGACTAACAGTAGTGGAAGCCATGACTTGTGGCCTGCCAACATTCGCTACTCGCCATGGTGGT

Y E A F G L T V V E A M T C G L P T F A T R H G G

2101 CCCAGGGAAATCATAGAGGATGGTGTTTCAGGATTCCATATTGATCCATATCACCCTGATAAGGCTGCTGATCTT

P R E I I E D G V S G F H I D P Y H P D K A A D L

2176 ATGGCAGAATTTTTTCAGAAGTGCACCGAGGATCCTGCCTACTGGGAGAAAATATCTGAAGGGGGTCTTCAGAGA

M A E F F Q K C T E D P A Y W E K I S E G G L Q R

2251 ATATATGAGAGGTATACGTGGAAGATCTACTCTGAAAGGCTCATGACACTGGCTGGAGTTTACAGTTTCTGGAAG

I Y E R Y T W K I Y S E R L M T L A G V Y S F W K

2326 TATGTTTCTAAACTTGAGAGGCGTGAAACGAGACGATATCTTGAGATGTTTTACATTCTAAAGTTCCGAGAATTG

Y V S K L E R R E T R R Y L E M F Y I L K F R E L

2401 GTCAAGTCTGTTCCGCTGGCAATCGATGAGGAGGCTACCGACGGTAAACATTAA

V K S V P L A I D E E A T D G K H *
