# Supplementary material for: Transcript profiling of sucrose synthase genes involved in sucrose metabolism among four carrot (Daucus carota L.) cultivars reveals distinct patterns
Source: BMC Plant Biol. 2018 Jan 5;18:8. doi: 10.1186/s12870-017-1221-1 (PMC5756371; doi:10.1186/s12870-017-1221-1)
Supplement: Supplementary file 3 — Nucleotide acid and deduced amino acid sequences of DcSus3 from carrot (DOC 34 kb) [file 12870_2017_1221_MOESM3_ESM.doc]

**Additional file 3:**

Fig S3: Nucleotide acid and deduced amino acid sequences of *DcSus3* from carrot.

1 ATGGATTCTGCGACAGCTGATAGCATGCCGGAGGCCTTGAGGCAGAGCCGGTACCATATGAAGAAATGTTTTTCG

M D S A T A D S M P E A L R Q S R Y H M K K C F S

76 AGATTTGTTGGAGGTGGGAGAAGATTGATGAAACATCAAACCTTGATGGATGAAATCGAAGAATCTATTGAGGAC

R F V G G G R R L M K H Q T L M D E I E E S I E D

151 AAGTCAGAAAGAAGCAAGGTTTTGGAAGGTTCACTTGGCCAAATCTTGAGCTCCACACAGGAGGCGGCTGTTAAT

K S E R S K V L E G S L G Q I L S S T Q E A A V N

226 CCACCTGATGTTGCTTTTGCTGTAAGACAAAATCCCGGTTGCTGGGAATATGTCAAGGTTAAAGCGAATGATCTG

P P D V A F A V R Q N P G C W E Y V K V K A N D L

301 TCTGTTGATGCCATCACTTCCACAGAGTACCTCAAGTTCAAGGAGCTGATATACGATGAAAAATGGGCAAATGAT

S V D A I T S T E Y L K F K E L I Y D E K W A N D

376 GATAATTCACTAGAAGTAGATTTTGGAGCATTTGATGTCACCACTCCTTGCCTAACCTTACCTTCTTCTATTGGA

D N S L E V D F G A F D V T T P C L T L P S S I G

451 AAAGGAGTTCATTTCATATCAAAGATCATGACAACTAAGTTCAGTGGAAATCCAGAAAGTGCAATGCCTTTGGTG

K G V H F I S K I M T T K F S G N P E S A M P L V

526 GAGTATTTGTTAGCTCTAAATCATCGAGGAGAGAATCTTATCATCAACGAGACTCTTAATACAGTTGCCAAGCTC

E Y L L A L N H R G E N L I I N E T L N T V A K L

601 CAGGCAGCATTACTGCTTGCTGATGTTTTCCTCTCCGTCCTTCCAAAAGATGCACCTTACCAGAATTTTGAGCAA

Q A A L L L A D V F L S V L P K D A P Y Q N F E Q

676 AAGCTTAAAGAGTGGGGATTCGAGAAAGGGTGGGGAGACACTACAGAAAGAGTTAGAGATACAATGAGGATAGTT

K L K E W G F E K G W G D T T E R V R D T M R I V

751 GCTGAGATACTACAGGCTCCAGATCCTATTAGCATGGAGTTGCTGTTTCAGAGGCTTCCGGTTACATTCAACGTG

A E I L Q A P D P I S M E L L F Q R L P V T F N V

826 GTAGTCCTCTCCATCCACGGGTATTTTGGCCAGTCAGATGTTCTTGGATTACCAGATACAGGAGGACAGGTGGTT

V V L S I H G Y F G Q S D V L G L P D T G G Q V V

901 TATATTCTAGACCAAGTAAAAGCTTTAGAGGAGGAACTGCTGCTCCGGATTAAGCAGCAAGGGTTGAGCATAAAG

Y I L D Q V K A L E E E L L L R I K Q Q G L S I K

976 CCTCAGATCATAGTGGTCACTCGTCTAATACCAGATGCTCAAGGTACAAAGTGCAATGTGGAGATGGAGCCTATT

P Q I I V V T R L I P D A Q G T K C N V E M E P I

1051 GACAACACCATGCATTCCCACATTCTTAGGATTCCGTTTAAAACTGACAACGGGATTCTAAAGCAATGGGTTTCT

D N T M H S H I L R I P F K T D N G I L K Q W V S

1126 AGGTTTGACATTTACCCTTTCTTGGAGAGGTTTTCACAGGATGCTACTGAAAAGATCCTGAAGTTGCTGGATTGT

R F D I Y P F L E R F S Q D A T E K I L K L L D C

1201 AAACCGGATCTTATAATAGGGAACTACACTGATGGAAATATTGTAGCATCCTTGATGGCTAGCAGACTAGGAGTA

K P D L I I G N Y T D G N I V A S L M A S R L G V

1276 ACCCAGGGCACCATTGCTCATGCTCTGGAGAAAACTAAGTACGAAGATTCTGATTTAAAATGGAAAGAGTTGGAT

T Q G T I A H A L E K T K Y E D S D L K W K E L D

1351 CCAAAATATCACTTCTCATGCCAGTTTATGGCTGATTTGATAGCGATGAATTCTGCTGATTTTATCATAACAAGC

P K Y H F S C Q F M A D L I A M N S A D F I I T S

1426 ACATATCAAGAAATTGCTGGAAGCAAGAAAAAAGCAGGACAATATGAAAGTCATGCAGCATTTACCATGCCAGGT

T Y Q E I A G S K K K A G Q Y E S H A A F T M P G

1501 CTCTGTAGAGTGGTATCAGGCATCAATGTCTTCGATCCAAAATTCAATATCGCTCCTCCAGGGGCTGAACAATCA

L C R V V S G I N V F D P K F N I A P P G A E Q S

1576 GTCTACTTTCCGTTCACAGAGAAAGAGAAGCGATTCAGCAAATTTCATTCCGCCATAGACCAATTGCTGTTCAGT

V Y F P F T E K E K R F S K F H S A I D Q L L F S

1651 AGAAATAATAACAATGAAGCCATCGGATTTCTAGCAAACAGGAAGAAACCGATAATATTTTCCATGGCGAGGCTC

R N N N N E A I G F L A N R K K P I I F S M A R L

1726 GATACAGTAAAGAACATTACTGGACTAGTGGAGATGTATGGAAAGAATAAGAGGCTCCAAAATTTGGTGAACCTG

D T V K N I T G L V E M Y G K N K R L Q N L V N L

1801 GTAATTGTTGCAGGCTTCTTTGATCCATCAAAATCTAAAGACAGAGAAGAAATGGTGGAAATAAATAAGATGCAT

V I V A G F F D P S K S K D R E E M V E I N K M H

1876 TCCTTGATAGAGAAGCACCAACTTGAGGGTCATGTCAGATGGATAGCAGCTCAAACTGACAGGTATCGTAACGGA

S L I E K H Q L E G H V R W I A A Q T D R Y R N G

1951 GAAATATACCGGTGCATTGCAGATACGAAAGGTGCTTTTGTGCAGCCTGCACTGTATGAAGCATTTGGGTTAACC

E I Y R C I A D T K G A F V Q P A L Y E A F G L T

2026 GTTATTGAAGCAATGAACTGTGGACTTCCTACCTTCGCGACAATTCACGGTGGACCAGCTGAGATTATTGTTGAC

V I E A M N C G L P T F A T I H G G P A E I I V D

2101 GGGATTTCAGGATTTCATATTGATCCTACAAAAGGAGAAGAATCAAGCAACAAGATTGCTGATTTCTTCGAAAAA

G I S G F H I D P T K G E E S S N K I A D F F E K

2176 TGCACCGTGGACAGAGAGTATTGGAACAGGCTGTCCCAGGATGCTTTGAAGCGTATTGATGAATGCTACACGTGG

C T V D R E Y W N R L S Q D A L K R I D E C Y T W

2251 AAGATCTATGCCAGCAATGTCCTGACAATGGGAGCCATCTATGGGGTTTGGAGACAGATGAATGAAGAAAAGAAG

K I Y A S N V L T M G A I Y G V W R Q M N E E K K

2326 CAAGCTAAGCAAAGATATATTGACATGTTCTATAGTCTCCAGTTCAGAAAATTGGCAAAAACTGTACGATTCACA

Q A K Q R Y I D M F Y S L Q F R K L A K T V R F T

2401 TTGGAGGAACCTTCGAAGTCTGCTATAGCAACGACCCTAAAACCTGAACAACCAACCCTCACAGTCGCAGAAGTA

L E E P S K S A I A T T L K P E Q P T L T V A E V

2476 CCGAGTCCTGTAGAACTGCCCAGACAAACCAATATAACACCAAGGAAAGATAAGCCACGACTCACTTTCCCCTCA

P S P V E L P R Q T N I T P R K D K P R L T F P S

2551 GATGAACCTGTAATTTGCCCCTGTTGGTGGTGGCTCCGTGTATGTCTGGCTTCCTTTATTACTGTCTACTGGCTC

D E P V I C P C W W W L R V C L A S F I T V Y W L

2626 ATGAAGATTGTAGCCTATACACGTGGCGACTAA

M K I V A Y T R G D *
