# Supplementary material for: Transcript profiling of sucrose synthase genes involved in sucrose metabolism among four carrot (Daucus carota L.) cultivars reveals distinct patterns
Source: BMC Plant Biol. 2018 Jan 5;18:8. doi: 10.1186/s12870-017-1221-1 (PMC5756371; doi:10.1186/s12870-017-1221-1)
Supplement: Supplementary file 4 — List of sucrose synthase gene sequences used in this study. (DOC 37 kb) [file 12870_2017_1221_MOESM4_ESM.doc]

**Additional file 4:**

Table S1: List of sucrose synthase gene sequences used in this study.

| Taxon | Sequence name | Accession number | Protein size |
| --- | --- | --- | --- |
| **Dicot plants**  *Gossypium hirsutum*  *Eucalyptus grandis*  *Solanum tuberosum*  *Citrus unshiu*  *Gossypium arboreum*  *Pisum sativum*  *Populus tomentosa*  *Populus trichocarapa*  *Eucalyptus grandis*  **Monocot plants**  *Zea mays*  *Bambusa oldhamii*  *Sorghum Bicolor*  *Triticum aestivum*  *Potamogeton distinctus*  *Hordeum vulgare*  *Oryza sativa*  *Prunus persica*  **Gymnosperms**  *Pinus taeda*  **C. Bacteria**  *Anabaena*  *Nostoc punctiforme* | GhSusy  EgSus3  StSus1  StSus2  StSus3  StSus4  StSus5  CuSuSy1  CuSuSy2  CuSuSyA  GaSus1  Gasus2  Gasus3  Gasus4  Gasus5  Gasus6  Gasus7  PsSuSy1  PsSuSy2  PsSuSy3  PsSuSy4  PtSuSy1  PtSuSy2  PtrSuSy1  PtrSuSy2  PtrSuSy3  PtrSuSy4  PtrSuSy5  PtrSUuS6  PtrSuSy7  EgSuSy3  ZmSus1  ZmSus2  ZmSus3  ZmSus4  ZmSus5  BoSuSy1  BoSuSy2  BoSuSy3  BoSuSy4  SbSus2  TaSuSy1  TaSuSy2  PdSUS2  HvSuSy1  HvSuSy2  OsSuSy1  OsSuSy3  OsSuSy4  OsSuSy7  PpSus2  PpSus3  PpSus4  PpSus5  PpSus6  PtaSuSy1  ASuSyA  NpSuSyA | U73588.2  DQ227994.1  M18745.1  AY205084.1  U24088.1  U24087.1  AJ537575.1  AB022092  AB029401  AB022091  JQ995522  JQ995523  JQ995524  JQ995525  JQ995526  JQ995527  JQ995528  AJ012080  AJ001071  AJ311496  AF079851  GU559727  GU559728  GU559729  GU559730  GU559731  GU559732  GU559733  GU559734  GU559735  DQ227994  L29418.1  L22296.1  BT069288.1  NM_001111941.2  EU971052.1  AF412036  AF412038  AF412037  AF412039  FJ513325  AJ001117  AJ000153  AB193516.1  X69931  Y15802  AK100334  AK100306  AK102158  HQ895725  KJ493331  KJ493332  KJ493333  KJ493334  KJ493335  EF619967  AJ010639  AJ316589 | 806  805  805  805  805  805  805  805  805  811  806  798  805  806  796  809  824  806  809  804  806  805  803  805  803  811  815  835  800  810  805  816  816  816  802  802  816  808  816  808  837  807  815  842  816  823  816  816  809  855  800  640  757  833  857  833  806  806 |
