# Supplementary material for: Transcript profiling of sucrose synthase genes involved in sucrose metabolism among four carrot (Daucus carota L.) cultivars reveals distinct patterns
Source: BMC Plant Biol. 2018 Jan 5;18:8. doi: 10.1186/s12870-017-1221-1 (PMC5756371; doi:10.1186/s12870-017-1221-1)

**Additional file 5:**

Fig S4: Results of RNA Electrophoresis

DC27 represents ‘Kurodagosun’;

BY represents ‘Baiyu’;

DC25 represents ‘Zizhou’;

SZ represents ‘Songzi’.


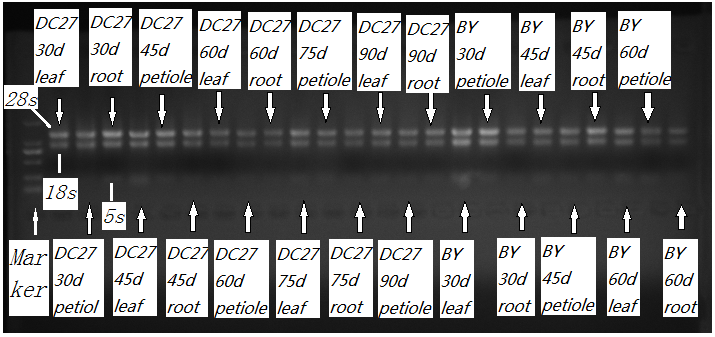


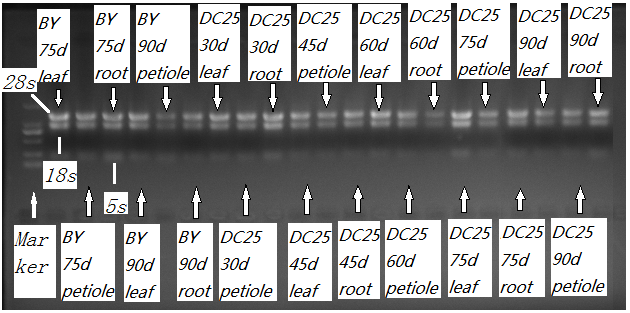


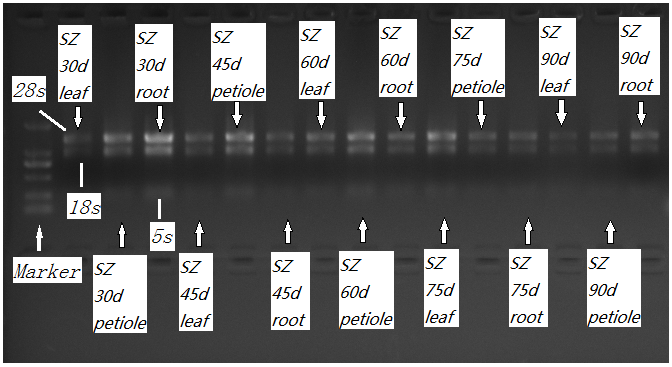

Supplement: Supplementary file 5 — Results of RNA Electrophoresis. DC27 represents ‘Kurodagosun’; BY represents ‘Baiyu’; DC25 represents ‘Zizhou’; SZ represents ‘Songzi’. (DOC 379 kb) [file 12870_2017_1221_MOESM5_ESM.doc]
