# Supplementary material for: Transcript profiling of sucrose synthase genes involved in sucrose metabolism among four carrot (Daucus carota L.) cultivars reveals distinct patterns
Source: BMC Plant Biol. 2018 Jan 5;18:8. doi: 10.1186/s12870-017-1221-1 (PMC5756371; doi:10.1186/s12870-017-1221-1)

**Additional file 6:**

Fig S5: Melting curves of *DcSus* genes related encoding sucrose synthase and two reference genes using in qPCR.


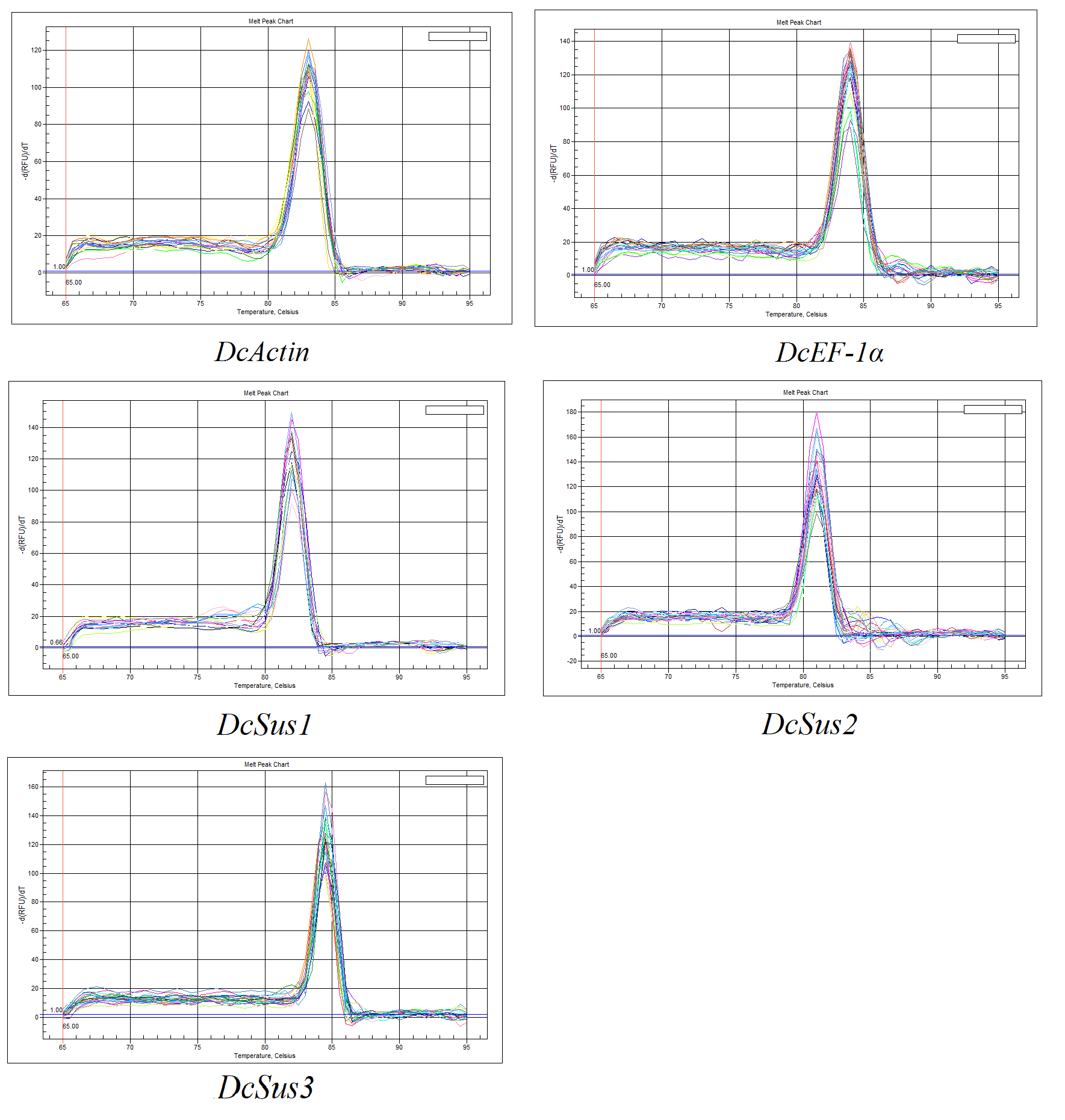

Supplement: Supplementary file 6 — Melting curves of DcSus genes related encoding sucrose synthase and two reference genes using in qPCR. (DOC 188 kb) [file 12870_2017_1221_MOESM6_ESM.doc]
