# Supplementary material for: Transcript profiling of sucrose synthase genes involved in sucrose metabolism among four carrot (Daucus carota L.) cultivars reveals distinct patterns
Source: BMC Plant Biol. 2018 Jan 5;18:8. doi: 10.1186/s12870-017-1221-1 (PMC5756371; doi:10.1186/s12870-017-1221-1)

**Additional file 7:**

Fig S6: Standard curves for *DcEF1-α, DcActin*, *DcSus1*, *DcSus2* and *DcSus3*. The linear correlation (*R^2^*) and PCR efficiencies (% E = (10^[−1/slope]^ - 1) × 100%) were calculated from the standard curve.





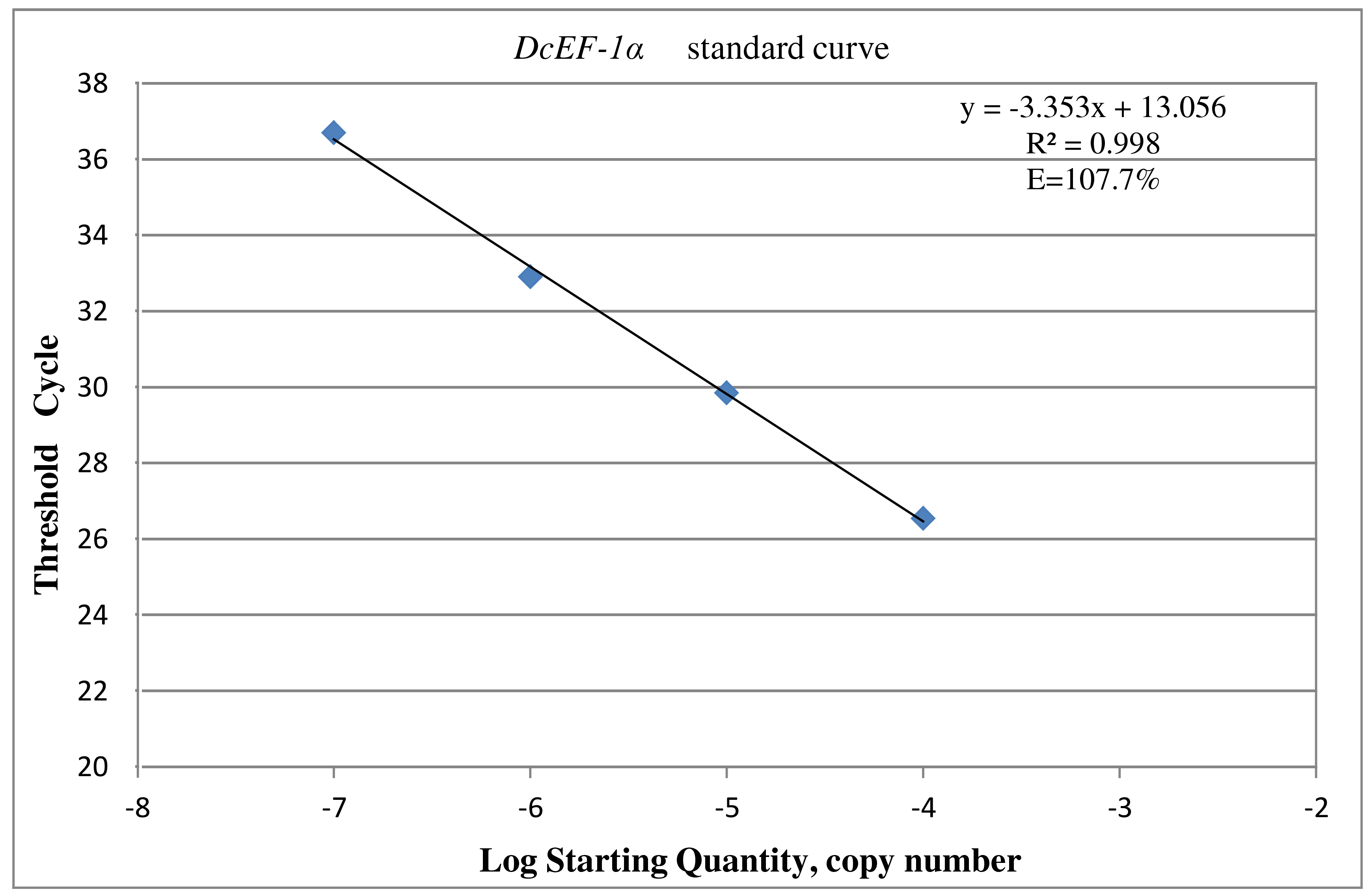

Supplement: Supplementary file 7 — Standard curves for DcEF1-α, DcActin, DcSus1, DcSus2 and DcSus3. The linear correlation (R2) and PCR efficiencies (% E = (10[−1/slope] - 1) × 100%) were calculated from the standard curve. (DOC 1194 kb) [file 12870_2017_1221_MOESM7_ESM.doc]
